# Central line associated blood stream infection (CLABSI) due to *Exophiala dermatitidis* in an adult patient: Case report and review

**DOI:** 10.1016/j.mmcr.2019.03.001

**Published:** 2019-03-02

**Authors:** Andrea Vila, Cintia Jahan, Cynthia Rivero, Claudio Amadio, Adela Ampuero, Hugo Pagella

**Affiliations:** a Hospital Italiano de Mendoza, San José, 3283, Argentina; b Obra Social Empleados Públicos (OSEP), Mendoza, 5500, Argentina; c Hospital Nestor Lencinas, Mendoza, 5500, Argentina

**Keywords:** *Exophiala dermatitidis*, Melanized fungi, Black yeast, Fungemia, *Wangiella dermatitidis*

## Abstract

*Exophiala dermatitidis* is a dematiaceous fungus with yeast-like and hyphal growth states that may cause cutaneous and visceral infections. Recently, E. dermatitidis has been linked to central line associated blood stream infection (CLABSI), probably due to its ability to produce extracellular polysaccharides and grow as biofilm. We describe an *E. dermatitidis* CLASBI. The strain was identified by morphological and molecular methods. *E. dermatitidis* CLASBI is highly uncommon, but seems to be increasing.

## 1 Introduction

*Exophiala dermatitidis*, previously known as *Wangiella dermatitidis*, is a dematiaceous, dimorphic fungus that may cause a large spectrum of human diseases [1,2]. As all phaeoid fungi (“phaeo” from the Greek meaning dark), *Exophiala* presents melanin on its cell wall [3], which enhances its survival in hostile environments [2]. Other aspects for pathogenicity include thermotolerance, adhesion, production of extracellular polysaccharide capsule, and biofilm formation [2]. Phenotypic identification of Exophiala spp. is hampered by its pleomorphic nature. Sequence data of the rDNA internal transcribed spacer (ITS) regions is reliable for species level diagnosis [4,5]. Additionally, proteomic identification using matrix assisted laser desorption/ionization time-of-flight mass spectrometry (MALDI-TOF MS) has been described as a useful method for identification of *E. dermatitidis* [6].

*E. dermatitidis* infections have been classified into superficial infections; cutaneous and subcutaneous diseases; and systemic deep-seated infections. The latter is not consistently accompanied by fungemia, is most often reported in Asia, approximately 70% of patients are immunocompromised, and the mortality rate is up to 80% [7]. It has been occasionally reported as outbreaks due to contaminated pharmaceutical products [8,9].

CLABSI due to *E. dermatitidis* has recently been described, therefore, its clinical manifestations and prognosis are not well known yet [10].

We describe a case of CLABSI due to a melanized yeast ultimately identified as *E. dermatitidis* by morphotype, ITS sequencing, and MALDI-TOF MS.

## 2 Case

A 75-years-old man was admitted due to respiratory failure (day 0). The patient had history of colonic and prostatic cancer 11 and 7 years earlier, respectively, both successfully treated without recurrence. He also had a history of hypertension, alcoholism, smoking and chronic obstructive pulmonary disease. During his transfer to the hospital in ambulance, he was given intravenous infusion of furosemide. Upon arrival his physical exam was significant for fever, cough, tachypnoea and tachycardia He was admitted to the intensive care unit (ICU), requiring mechanical ventilation. Laboratory was remarkable for a white blood cell count of 10.5/mm3, C-reactive protein, lactate dehydrogenase and lactic acid were increased. An initial lung CT angiography showed bilateral ground glass pulmonary infiltrate without evidence of pulmonary embolism. With presumptive diagnosis of community acquired pneumonia, bronchoalveolar lavage (BAL) and BCs were done (Bactec aerobic medium; BD Diagnostic Instrument Systems; Bactec 9240). Fungal and bacterial stains and cultures from BAL were negative, and Galactomannan (PlateliaTM Aspergillus Ag) OD index was 0,45. Patient was started on piperacillin-tazobactam, vancomycin and hydrocortisone, and become afebrile at 24 hs.

On day 4, BCs were negative, thus vancomycin was discontinued. On day 6, after a short period of stabilization, he became hypotensive requiring inotropic assistance. Three BCs (1 from peripheral vein and 2 from CVC) were taken. A transthoracic echocardiogram didn't show valvular lesions.

On day 8 abdominal laparoscopy was done due to ascites. Ascitic fluid culture was negative. During the procedure a liver biopsy was done, later showing cirrhosis.

**Fig. 1:**
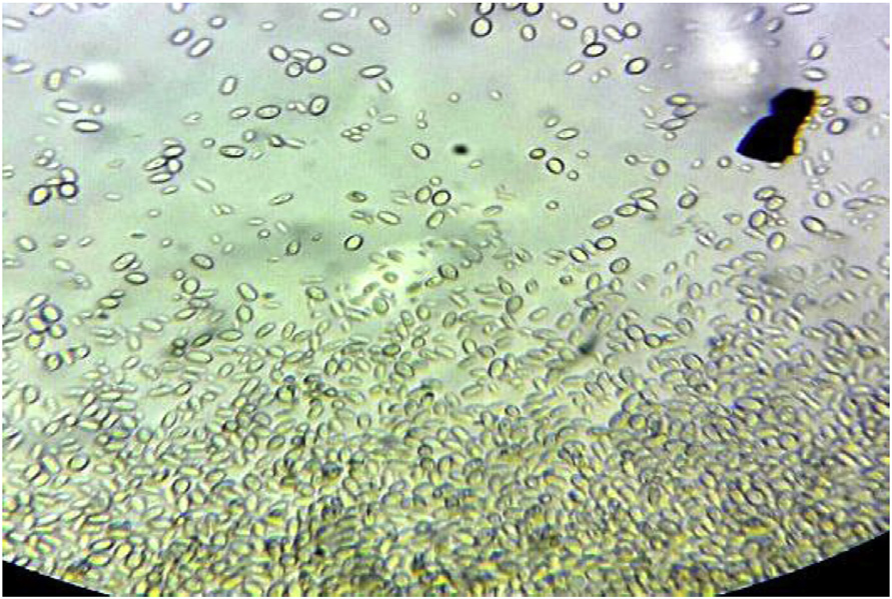
Blood Culture fresh microscopy.

**Fig. 2:**
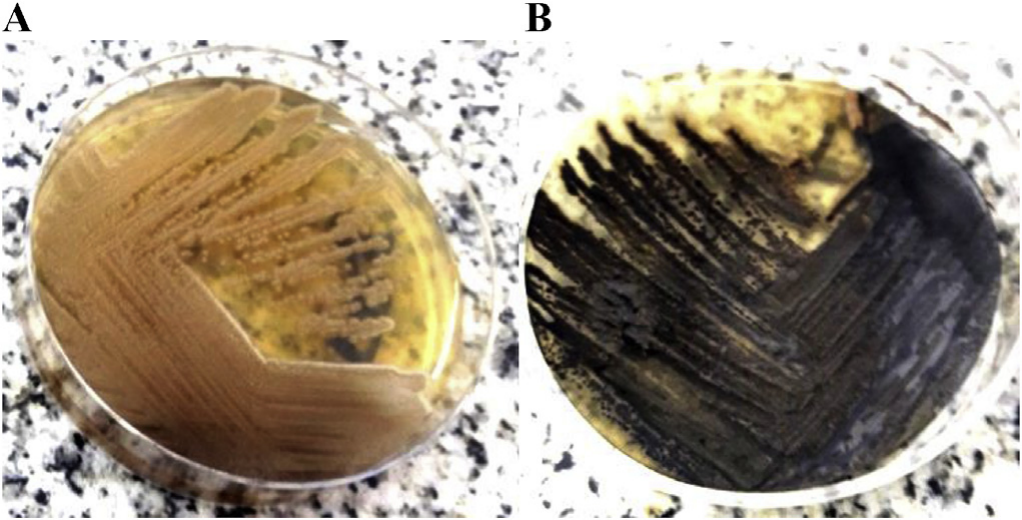
Culture on Sabouraud agar at 37 °C. A. Day 3: slow growing of smooth mucoid yeast-like colonies. B. After 7 days: black raised velvet colonies, suede-like in texture, olivaceous-grey with the development of aerial mycelium. Reverse is olivaceous-black.

**Fig. 3:**
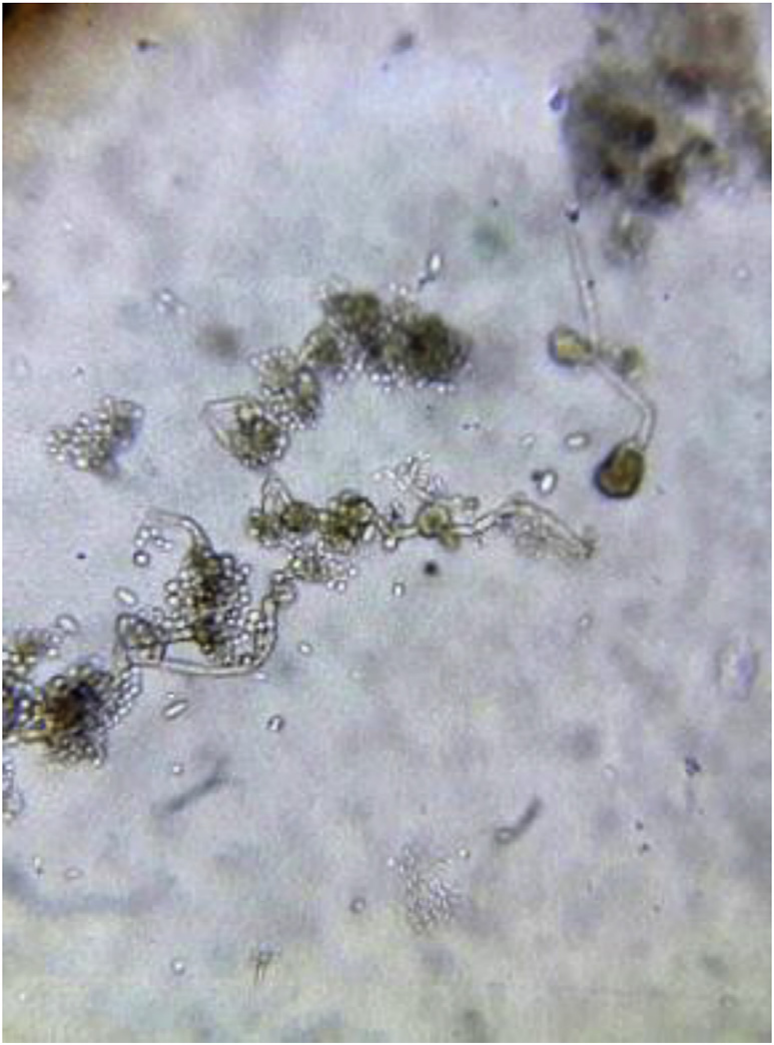
Microscopic examination of culture. Hyaline budding yeast-like cells, ovoid to elliptical, with thin wall. Pigmented (dematiaceous), septate and branched hyphae, with annellidic conidiophores that produce round to ovoid conidia of different sizes, with a thin smooth wall, that accumulate in slimy balls at the apices of the annelids or down their sides forming mucosal aggregates.

On day 9 the pair of BCs taken at day 6 showed a positive growth index. Direct microscopy showed hyaline, ovoid to elliptical yeasts (Fig. 1). A new set of BCs were drawn in order to confirm that finding. On day 13, yeasts were found on all BCs samples from days 6 and 9. The patient denied outdoor activity or recent traveling. No skin lesions were found on physical exam, and the portal of entry remained unclear. After 3 days, subcultures on Sabouraud agar showed slow-growing colonies, initially with smooth glossy mucous appearance, that over time became velvety olivaceous black (Fig. 2 A, B). Microscopy revealed pigmented septate branched hyphae with annelidic conidiogenesis, and ellipsoidal conidia of different sizes with a thin wall, forming aggregates (Fig. 3). The isolate was identified as *Exophiala* spp. MALDI-TOF (Bruker Daltonics) identified the colonies as *E. dermatitidis* with a 1.689 score. Patient was diagnosed of CLASBI due to non-Candida fungus according to CDC definition. Anidulafungin was started and CVC was removed. The strain was submitted to the national mycology reference center (“Departamento de Micología, Instituto Nacional de Enfermedades Infecciosas Dr. Carlos G. Malbrán”), for further for molecular identification. Sequence data of the rDNA ITS regions of the D1-D2 of the large (28S) ribosomal subunit of the isolate resulted in 98.6% similarity to *E. dermatitidis*. Antifungal susceptibility testing was performed according the methodology recommended by the CLSI, document M38-A2 (2008), revealing a MIC (μg/mL) for amphotericin B, anidulafungin, and caspofungin of 0.125, 0,008 and 0,008 respectively.

Neither renal ultrasound nor ophthalmologic examination, reveal evidence of disseminated fungal infection. Standard histological stains for fungi were requested on liver histological sample with negative results. Histological diagnosis of cirrhosis was done.

BCs done on day 16 were negative. On day 18 the patient died due to haemoptysis and supraventricular tachycardia.

Due to described outbreaks of *Exophiala* spp. caused by medication contamination, audit and surveillance of the practices of preparation and administration of intravenous medication was made. No irregularities were found. ICU drug preparation surfaces were cultured in search for fungi, which were negative for *Exophiala* spp. Surveillance for secondary cases in ICU patients was conducted by incubated all BCs in a prolonged manner (14 days) during a period of 3 months from the index case. No other patient presented *E. dermatitidis* fungemia.

## 3 Discussion

Melanized fungi cause a diverse range of diseases. Although traditionally associated with chronic cutaneous and subcutaneous infections, recently, there seems to be an increasing frequency of systemic forms [3]. The genus *Exophiala*, order *Chaetotheriales*, class *Ascomycota* [3], contains about 40 species [11]. *E. dermatitidis* is a polyextremotolerant, oligotrophic, metabolically versatile fungus, with noteworthy surviving strategies, and enormous ecological plasticity, capable of survive nutritionally poor or toxic habitats. By modifying its morphology and behavior, *E. dermatitidis* may move from natural to human habitat and to the human host [11].

Although usually named as a ¨yeast¨, *E. dermatitidis* is polymorphic, thus, able to produce various morphological structures (black yeasts, hyphae, and sclerotic bodies) [3,12]. It has been named as dimorphic due to its capacity of changing between waterborne yeast (submersed growth), and filamentous stage (hydrophobic hyphal growth), this through a yeast-hyphal switch in simultaneously or consecutively occurring phases [1,2,13]. Dried fungal morphotypes (mycelia and conidia) are able of surviving repeated exposures to 60–80 °C. When *E. dermatitidis* gets in an aquatic medium (tap water, pharmaceutical products, bloodstream), far from dying, it develops the submerged morphotype (yeasts), which is able to grow at temperatures of 37 °C, produce extracellular polysaccharides around yeast cells (capsule) [11], and grow in a biofilm mode [8]. In the human host, the yeast form is able to disseminate hematogenously; while the hyphal growth usually causes localized infection [2]. Melanin is a complex polymer deposited in the cell wall, that enhances the survival of fungi in hostile environments, conferring them resistance to multiple ecological and biological threats, including phagocytosis [2]. It also contributes to the organism's ability to elude host immunity through blocking the effects of hydrolytic enzymes and free radicals liberated by phagocytic cells [2,4]. *E. dermatitidis* has been isolated in various human-made environments, such as dishwashers, saunas and steam baths [13,14]. Also, gastrointestinal tract (GIT) asymptomatic carriage, as well as colonization of the respiratory tract in cystic fibrosis patients have been described [2,15].

Due to its remarkable adaptive plasticity, *E. dermatitidis* is capable of causing a wide range of infectious diseases, depending on the route and mode of entry, and the host immunological condition.

Cutaneous and subcutaneous infections usually take place throughout direct entry through the skin, but the route of infection of disseminated cases is not always ascertained [2]. When *E. dermatitidis* gets into the bloodstream, it may cause systemic, disseminated, often neurotropic infections with deep organ involvement (endocarditis, pneumonia and particularly brain abscess). Invasive *E. dermatitidis* disease is rare and most cases have been reported in Asia [2], but may occur in outbreaks due to direct iatrogenic inoculation. In 2002 an outbreak of *E. dermatitidis* meningitis or arthritis related to epidural or intra-articular injections of contaminated methylprednisolone acetate was described [9].

Recently described cases of CLASBI due to *E. dermatitidis* are even more unusual [7]. When bloodstream invasion occurs through a CVC, *E. dermatitidis* finds the opportunity to adhere, produce extracellular matrix and grow as biofilm, which protects the fungus against host defenses and anti-infective agents [1].

Outbreaks of CLASBI due to intravenous infusion of fungus-contaminated medication through CVC have been described. In an outbreak at an oncology clinic [8], despite the fact that all patients were exposed to a contaminated intravenous solution, only those with CVC developed infection [8].

To our knowledge there have been 6 cases reported of sporadic (not outbreak-related) CLASBI due to *E. dermatitidis* in adult patients [7,10,[16], [17], [18], [19]] (Table 1). Five of these patients had neoplasia, and all the patients had CVC for chemotherapy or nutritional support [7]. The presence of a CVC may be necessary for E. dermatitidis to establish bloodstream infection. Our patient did not have neoplastic active disease. He was a critical care patient with CVC.

**Table 1:** Summary of published cases of *E. dermatitidis* CLASBI in adults excluding outbreaks.

Author Country Year Reference	Sex Age	Comorbidities at time of diagnosis	Site of isolation Diagnostic method	Susceptibility MIC (μg/mL)	Treatment	Outcome
Simpson UK 1995 [1]	F 53	CVC Parenteral nutrition Prolonged antibiotic administration	2 CVC-BC Morphotype	Not done	CVC removal Fluconazole 4 days	Survived
Larocco USA 2002 [2]	F 61	Metastatic breast cancer Chemotherapy TI-CVAD	1 P-BC Morphotype	Not done	CVC removal ABD followed by itraconazole 8 weeks	Survived
Tseng Taiwan 2005 [3]	F 52	Metastatic lung cancer Chemotherapy TI-CVAD	2 CVC-BC Morphological and biochemical properties	Fluconazole 48 AB 0.19	CVC removal ABD 17 days	Died
Chalkias USA 2014 [4]	M 57	Relapsed lymphoma HSCT GVHD PICC	5 pairs of P-BC* Morphological and biochemical properties	Fluconazole 8 Itraconazole 0.25 Voriconazole 0.125 Posaconazole 0.125 AB 1; 5-FC > 64 Caspofungin 8 Anidulafungin 8 Micafungin 8 TRF 0.06	CVC removal Voriconazole and L-AB 10 days	Died
Kakuya Japan 2014 [5]	F 47	Metastatic lingual and esophageal cancer Malnutrition Chemotherapy TI-CVAD	TI-CVAD culture Morphotype	Mica >16	CVC removal Micafungin 12 days	Died
Watanabe Japan 2018 [6]	M, 45	Myelofibrosis Acute myeloid leukemia UCBT Prolonged neutropenia	P-BC*	AB: 0.5	CVC removal L-AB until dead	Died
			Morphotype	MCFG: > 16		
			MALDI-TOF	FLCZ: 16		
Our case	M, 75	Cirrhosis COPD CVC	6 BCs Morphotype MALDI-TOF ITS sequencing	AB 0.125 Caspo 0.008 Anidula 0.008	CVC removal Anidulafungin until dead	Dead

F: female; M: male; CVC: central venous catheter; BC: blood-culture; P: peripheral; TI-CVAD: totally implanted central venous access device; AB: amphotericin B; 5-FC: 5-flucytosine; HSCT: hematopoietic stem cell transplant; GVHD: graft-versus-host disease; PICC: peripherally inserted central line; L-AB: liposomal amphotericin; TRF: terbinafine; UCBT: umbilical cord blood transplantation; COPD: chronic obstructive pulmonary disease; ITS: internal transcribed spacer.

*Note: fungemia while on mica prophylaxis.

*E. dermatitidis* might be underestimated as etiologic agent of CLASBI owing to it slow growth [15]. Therefore, in ICU patients with suspected CLASBI without microbiological diagnosis, prolonged incubation of BCs may be useful. Rare invasive fungal infections should lead to consider unusual sources of exposition and windows of opportunity for microbial growth [11]. The source and route of infection in our case remains elusive. There were no other cases that could suggest the existence of contaminated infusion, and *E. dermatitidis* was not documented in the ICU surfaces that were sampled. Although prolonged incubation of BC from all ICU patients was done during a 3 months period, in order to detect a possible hospital outbreak due to *E. dermatitidis*, there were no additional further cases. The patient did not present cutaneous lesions; but he had the received intravenous infusion in an ambulance, which could not be tracked. *E. dermatitidis* has been found in GIT and fungemia in seriously ill patients may occur due to translocation of fungi from edematous or ischemic segments of GIT [13,19]. We didn't perform culture for fungi in feces. We consider that the fungus could have entered bloodstream during the intravenous infusion received in the ambulance, or by translocation from GIT. In any case, it has been shown that 92% of clinical isolates of *E. dermatitidis* exhibit biofilm formation [1]. The CVC may have favored adherence, biofilm formation and persistence of fungemia.

This has been the first case of bloodstream isolation of *Exophiala* spp. in our Hospital.

Although *Exophiala* can be identified to the genus level by morphological characteristics, species identification must be complemented by molecular tests [19]. Sequencing of the ITS regions of rDNA is recommended [2,5]. Although MALDI-TOF MS has been described as an accurate method for identification of *E. dermatitidis* [14], in our case the identification score was found within the “not reliable” species match range (bellow 1.7).

*E. dermatitidis* has unique problems when preparing an inoculum (both the yeast and mould phase are present within the same colony resulting in a mixed population) [5]. Also, the ideal reading time is unknown due to their low growth rate [5]. There are no established MIC breakpoints to interpret susceptibility results in a standardized fashion [19], and information about sensitivity of *E. dermatitidis* is very limited and shows a very broad MIC interval (MIC range, mg/L), specially for echinocandins: terbinafine (0.06–0.5); amphotericin b (0.01–1); itraconazole (0.03–0.5); voriconazole (0.06–1); posaconazole (0.016–0.25); isavuconaloze (0.03–1); fluconazole (2–32); echinocandins (0.03–16) [5,6,19]. Terbinafine shows the best *in vitro* activity, while 5-flucytosine displays very high MICs (range 8–64 mg/L) [5,19].

We suggest susceptibility testing to guide treatment in order to choose the drug with the lowest MIC and good penetration at the infection site. Although monotherapy with an echinocandin does not seems to be optimal, the MIC to anidulafungin in our strain was notably low (0,008 mg/L), the patient didn't have evidence of deep organ seated infection, and echinocandins show good activity against biofilms, thus we didn't change therapy. Colistin has shown *in vitro* activity against *Exophiala* spp [19] and may be useful combined with antifungals for treating fungal biofilm-related infections, such as CLASBI [1]. Contrasting previous reports, in our case echinocandins MIC was low, and seems to be the lowest MIC described in clinical isolates.

To our knowledge, this is the first reported case of *E. dermatitidis* CLASBI in Argentina. *E. dermatitidis* seems to be expanding its borders beyond Asia. Although no definite treatment guidelines have been proposed for the management of *E. dermatitidis* infections, in case of CLASBI, immediate removal of CVC and targeted antifungal therapy guided by MIC, are key for improving outcome [7].

## Conflict of interest

Authors have no conflict of interest. The authors have obtained written and signed consent to publish the case report from the patient's legal guardian.

## Funding

This research did not receive any specific grant from funding agencies in the public, commercial, or not-for-profit sectors.

## References

[1] Kirchhoff L., Olsowski M., Zilmans K., Dittmer S., Haase G., Sedlacek L. (2017). Biofilm formation of the black yeast-like fungus *Exophiala dermatitidis* and its susceptibility to antiinfective agents. Sci. Rep..

[2] Seyedmousavi S., Netea M.G., Mouton J.W., Melchers W.J., Verweij P.E., de Hoog G.S. (2014). Black yeasts and their filamentous relatives: principles of pathogenesis and host defense. Clin. Microbiol. Rev..

[3] Chen Z., Martinez D.A., Gujja S., Sykes S.M., Zeng Q., Szaniszlo P.J. (2014). Comparative genomic and transcriptomic analysis of *Wangiella dermatitidis*, a major cause of phaeohyphomycosis and a model black yeast human pathogen. G3 (Bethesda).

[4] Revankar S.G., Sutton D.A. (2010). Melanized fungi in human disease. Clin. Microbiol. Rev..

[5] Silva W.C., Goncalves S.S., Santos D.W., Padovan A.C., Bizerra F.C., Melo A.S. (2017). Species diversity, antifungal susceptibility and phenotypic and genotypic characterisation of *Exophiala spp.* infecting patients in different medical centres in Brazil. Mycoses.

[6] Kondori N., Erhard M., Welinder-Olsson C., Groenewald M., Verkley G., Moore E.R. (2015). Analyses of black fungi by matrix-assisted laser desorption/ionization time-of-flight mass spectrometry (MALDI-TOF MS): species-level identification of clinical isolates of *Exophiala dermatitidis*. FEMS Microbiol. Lett..

[7] Tseng P.H., Lee P., Tsai T.H., Hsueh P.R. (2005). Central venous catheter-associated fungemia due to *Wangiella dermatitidis*. J. Formos. Med. Assoc. = Taiwan yi zhi.

[8] Vasquez A., Zavasky D., Chow N.A., Gade L., Zlatanic E., Elkind S. (2018). Management of an outbreak of *Exophiala dermatitidis* bloodstream infections at an outpatient oncology clinic. Clin. Infect. Dis. Off. Publ. Infect. Dis. Soc. Am..

[9] Centers for Disease C Prevention (2002). *Exophiala* infection from contaminated injectable steroids prepared by a compounding pharmacy--United States, July-November 2002. MMWR Morb. Mortal. Wkly. Rep..

[10] Nachman S., Alpan O., Malowitz R., Spitzer E.D. (1996). Catheter-associated fungemia due to *Wangiella (Exophiala) dermatitidis*. J. Clin. Microbiol..

[11] Song Y., Laureijssen-van de Sande W.W.J., Moreno L.F., Gerrits van den Ende B., Li R., de Hoog S. (2017). Comparative ecology of capsular Exophiala species causing disseminated infection in humans. Front. Microbiol..

[12] Tesei D., Marzban G., Marchetti-Deschmann M., Tafer H., Arcalis E., Sterflinger K. (2015). Proteome of tolerance fine-tuning in the human pathogen black yeast *Exophiala dermatitidis*. J. Proteom..

[13] Sudhadham M., Prakitsin S., Sivichai S., Chaiyarat R., Dorrestein G.M., Menken S.B. (2008). The neurotropic black yeast *Exophiala dermatitidis* has a possible origin in the tropical rain forest. Stud. Mycol..

[14] Borman A.M., Fraser M., Szekely A., Larcombe D.E., Johnson E.M. (2017). Rapid identification of clinically relevant members of the genus Exophiala by matrix-assisted laser desorption ionization-time of flight mass spectrometry and description of two novel species, *Exophiala campbellii* and *Exophiala lavatrina*. J. Clin. Microbiol..

[15] Chowdhary A., Perfect J., de Hoog G.S. (2014). Black molds and melanized yeasts pathogenic to humans. Cold Spring Harb. Perspect. Med..

[16] Simpson A.J., Nightingale J.M. (1995). Intravascular line infection with *Exophiala dermatitidis*. Lancet.

[17] LaRocco A., Netzer R.C. (2002). Central venous catheters as a risk factor for disseminated phaeohyphomycosis?. Clin. Infect. Dis.: Off. Publ. Infect. Dis. Soc. Am..

[18] Watanabe N., Gotoh A., Shirane S., Hamano Y., Hirai Y., Shimizu M. (2018). Breakthrough *Exophiala dermatitidis* infection during prophylactic administration of micafungin during second umbilical cord blood transplantation after graft failure. Transpl. Infect. Dis..

[19] Chalkias S., Alonso C.D., Levine J.D., Wong M.T. (2014). Emerging pathogen in immunocompromised hosts: *Exophiala dermatitidis* mycosis in graft-versus-host disease. Transpl. Infect. Dis..

